# Supplementary material for: Carbon Nanotube-Mediated Delivery of PTEN Variants: In Vitro Antitumor Activity in Breast Cancer Cells
Source: Molecules. 2024 Jun 11;29(12):2785. doi: 10.3390/molecules29122785 (PMC11206347; doi:10.3390/molecules29122785)
Supplement: Supplementary file 1 [file molecules-29-02785-s001.zip › molecules-3014699-supplementary.pdf]

Carbon Nanotube-Mediated Delivery of PTEN Variants: In Vitro Antitumor Activity in Breast Cancer Cells

Rigini M. Papi [1,*,†], Konstantinos S. Tasioulis [1,†], Petros V. Kechagioglou [1,‡], Maria A. Papaioannou [2], Eleftherios G. Andriotis [3] and Dimitrios A. Kyriakidis [1]

[1] Laboratory of Biochemistry, Department of Chemistry, Aristotle University of Thessaloniki, 541 24 Thessaloniki, Greece; kstasioul@chem.auth.gr (K.S.T.); petran_k@hotmail.gr (P.V.K.); kyr@chem.auth.gr (D.A.K.)
[2] Laboratory of Biological Chemistry, School of Medicine, Aristotle University of Thessaloniki, 541 24 Thessaloniki, Greece; mpapaioannou@auth.gr
[3] Laboratory of Organic Chemical Technology, Department of Chemistry, Aristotle University of Thessaloniki, 541 24 Thessaloniki, Greece; e.andriotis@yahoo.gr
* Correspondence: rigini@chem.auth.gr; Tel.:+30-2310997878
† These authors contributed equally to this work.
‡ Current address: Cell Biology Unit, University Medical Center, 55128 Mainz, Germany.

Supplementary Materials

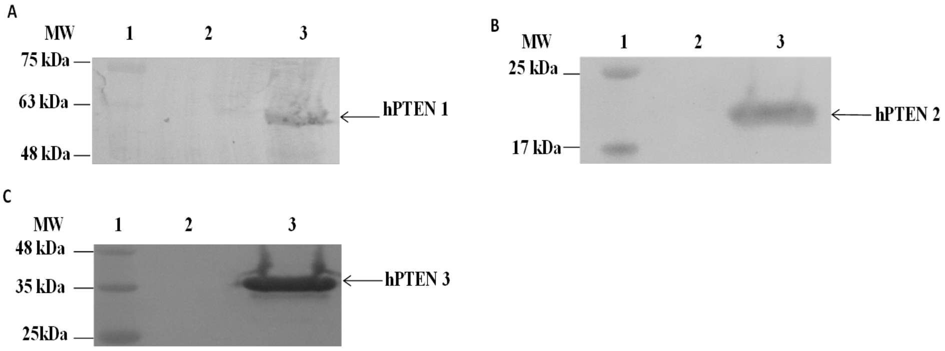

Figure S1. Immunostaining of the recombinant proteins (A) hPTEN1, (B) hPTEN2 and (C) hPTEN3 with anti-His-Tag (1:1000, Cell Signaling Technology). Total cells from 1 mL of bacterial culture, before (Lane 2) and after (Lane 3) the 1 mM IPTG induction, were electrophoresed on a 10% (w/v) SDS-PAGE and transferred onto a nitrocellulose membrane. Lane 1 presents the pre-stained protein markers.

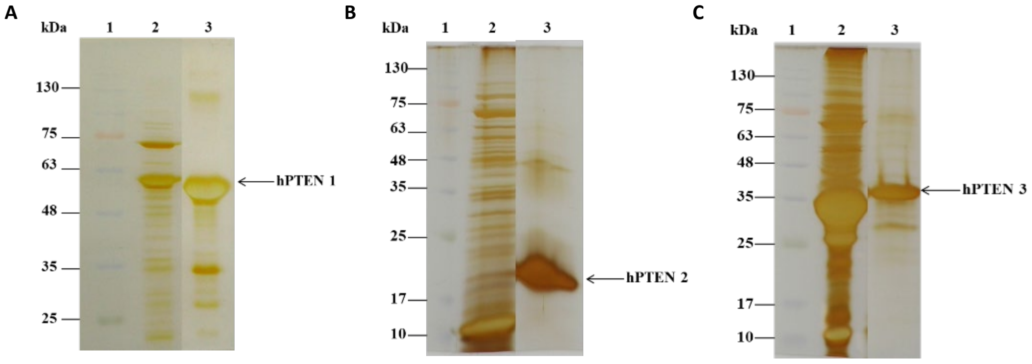

Figure S2. Isolation of the recombinant proteins (**A**) hPTEN1, (**B**) hPTEN2 and (**C**) hPTEN3, using Ni-TED affinity chromatography, under denatured conditions. SDS-PAGE on 10% (w/v) polyacrylamide

gels and silver nitrate staining of 50 μg inclusion bodies protein content (Lane 2) and 50 μg of each eluent III with 250 mM imidazole (Lane 3). Lane 1 represents the pre-stained protein markers.

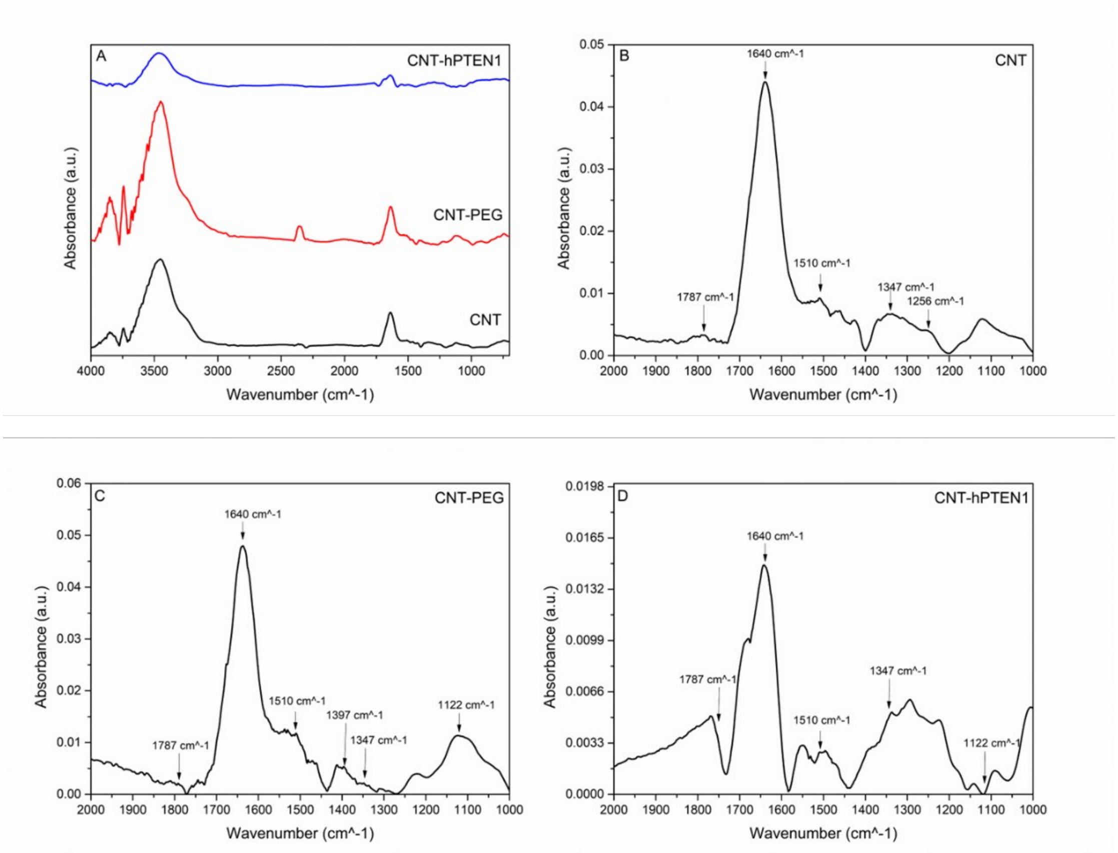

Figure S3. (**A**) FTIR analysis was applied to verify the immobilization of the proteins on CNTs. IR spectra in the range of 1000 – 2000 cm^{-1} wavenumber of (**B**) the oxidized carbon nanotubes (CNT), (**C**) the PEGylated carbon nanotubes (CNT-PEG), and (**D**) the hPTEN1 protein biofunctionalized CNTs (CNT-hPTEN1).

FTIR analysis was applied to verify the immobilization of the proteins on carbon nanotubes (Figure S3 – data here shown only for hPTEN1, similar data were obtained for hPTEN2 and hPTEN3). The FTIR spectra obtained for the oxidized carbon nanotubes (CNT), PEGylated carbon nanotubes (CNT-PEG), and hPTEN1 functionalized carbon nanotubes (CNT-hPTEN1) are presented in Figures 3B, C, and D, respectively. Based on the spectra analysis, the peak around 1787 cm^{-1} is attributed to C=O stretching vibration of the carboxylic acid group of both nanotubes and protein. The stretching vibration of the C=C bond around 1640 cm^{-1} is a characteristic peak of carbon nanotubes. The peak located at 1510 cm^{-1} is assigned to the bending vibration of N-H bond of the associated protein secondary amides and appears as a distinct peak in the CNTs-hPTEN1 spectrum. On the other hand, the two peaks at 1347 cm^{-1} and 1256 cm^{-1} correspond to the stretching and bending vibration of carbon nanotubes C-O bond, respectively. PEGylated modified nanotubes exhibit a peak at 1397 cm^{-1} corresponding to C-O stretching vibration and one at 1122 cm^{-1} which is characteristic of the C-N bond stretching vibration of primary amines, that does not exist in the CNT-hPTEN1 spectrum, indicating the immobilization of hPTEN1 on the PEGylated carbon nanotubes

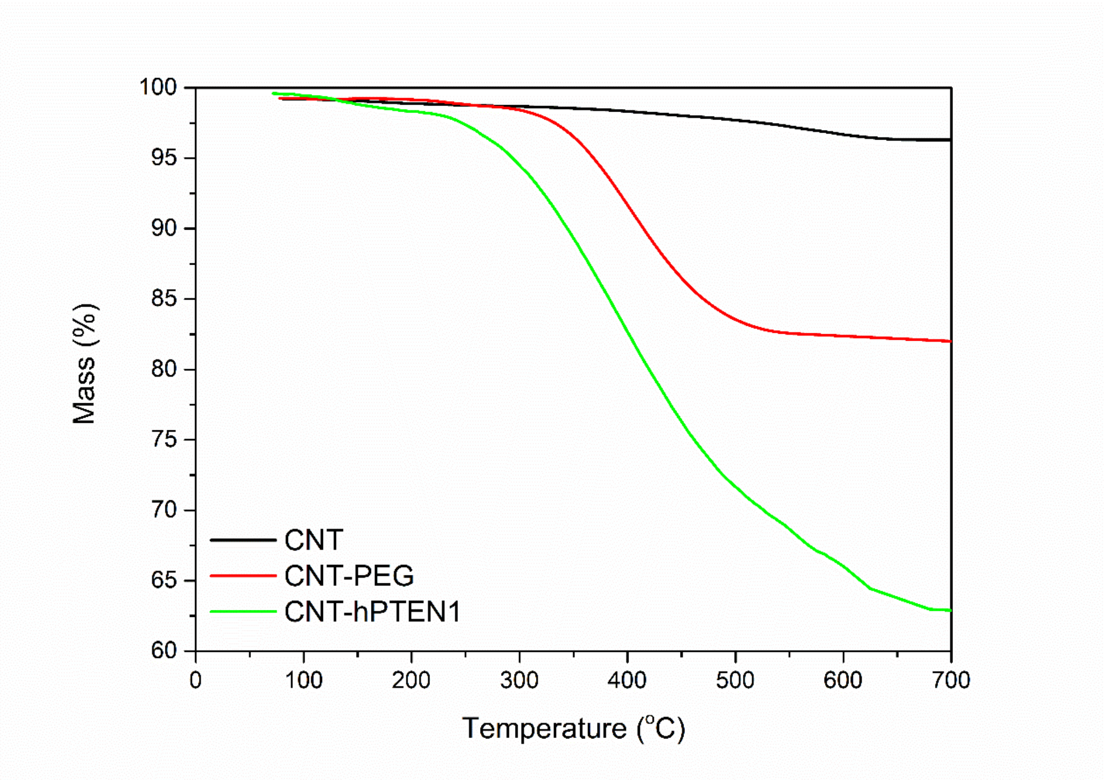

Figure S4. Thermogravimetric analysis (TGA) of (i) the oxidized carbon nanotubes (CNT) (blue color line), (ii) the PEGylated carbon nanotubes (CNT-PEG) (red color line) and (iii) the hPTEN1 protein biofunctionalized carbon nanotubes (CNT-hPTEN1) (green color line).

Furthermore, Figure S4 explains the thermogravimetric analysis of the hPTEN1 immobilized onto the CNTs. A decrease in CNTs mass was noticed with increasing temperature. The weight loss of the oxidized CNTs (4.7%) is accredited to the TGA-induced breakdown of the carboxylic acid groups of CNTs, while the further weight loss (18%) in the case of the CNTs-PEG suggested the presence of PEG on their surface. In addition, the additional weight loss (37.2%) observed in the case of the CNTs-hPTEN1, reflected the effective biofunctionalization, especially considering that only 5% of the CNTs was oxidized and thus PEGylated.

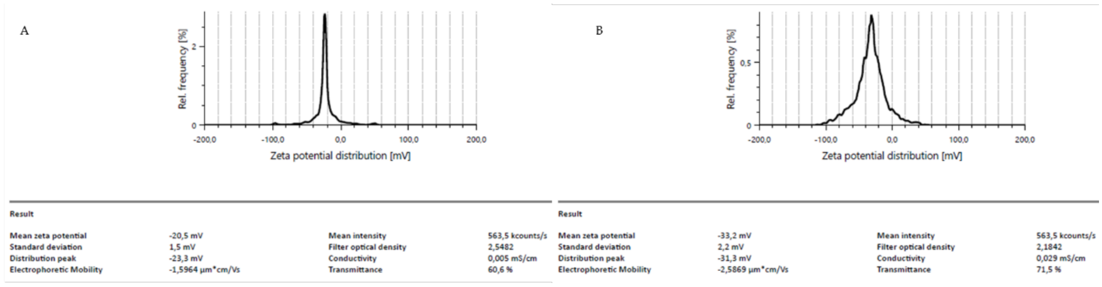

Figure S5. Zeta-potential (mV). The zeta-potential of (A) PEGylated CNTs was (-20.50 mV), while (B) the PTEN-conjugated CNTs (CNTs-hPTEN1) exhibited lower zeta-potential (-33.20 mV).

Finally, carbon nanotubes functionalization was characterized by measuring their zeta potential. Zeta potential quantifies nanoparticles' surface charge. As resulted (Figure S5), protein biofunctionalized CNTs exhibited slightly lower surface zeta-potential (-33.20 mV) compared to the control, PEGylated CNTs (-20.50 mV) under physiologic conditions, implying the efficient surface immobilization of the protein onto the CNTs, owing to the presence of the protein carboxyl groups on the particles surface.

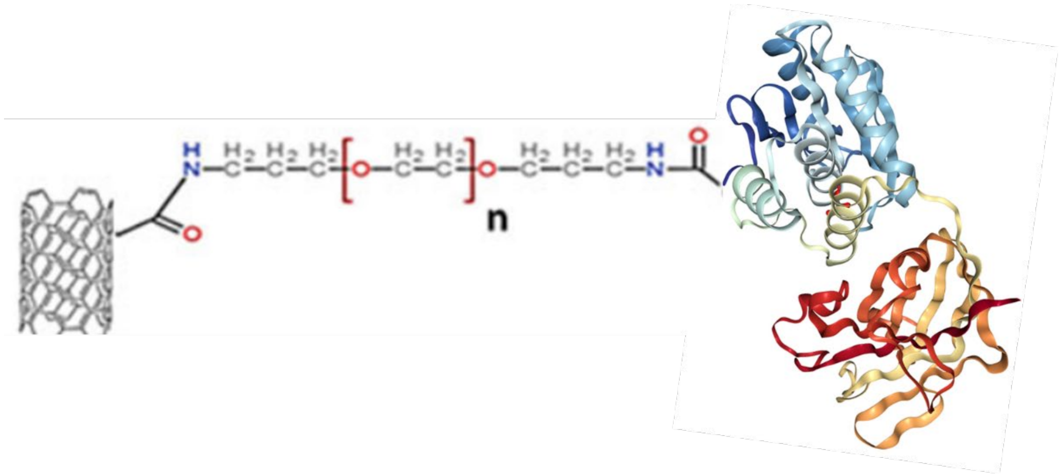

Figure S6. Chemical structure of biofunctionalized CNTs-hPTEN1. Reproduced after modification from [57].

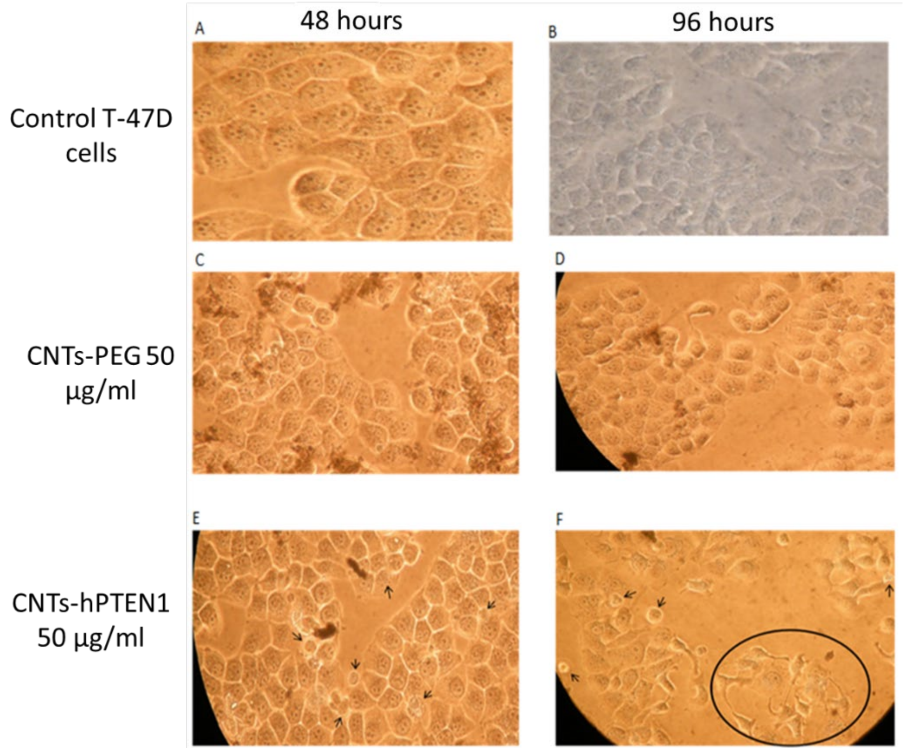

Figure S7. T-47D cells morphology after incubation with hPTEN1 biofunctionalized CNTs. T-47D cells after (**A**) 48 and (**B**) 96 hours without treatment, and after incubation with 50 μg/mL of (**C**) PEGylated CNTs (48 hours), (**D**) PEGylated CNTs (96 hours), (**E**) CNTs-hPTEN1 (48 hours) and (**F**) CNTs-hPTEN1 (96 hours). Cell photos were taken with a Nikon optical microscope at 200X magnification.

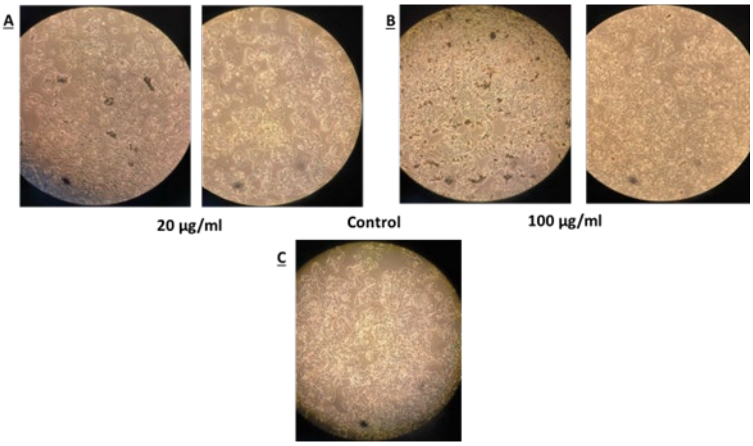

Figure S8. ZR-75-1 cells morphology after incubation for 72 hours with CNTs-PEG or CNTs-hPTEN1 at a final concentration of 20 µg/ml (**A**), and 100 µg/ml (**B**). Control ZR-75-1 cells (**C**) also harvested 72 hours after their seeding. Cell photos were taken with a Nikon optical microscope at 100X magnification.

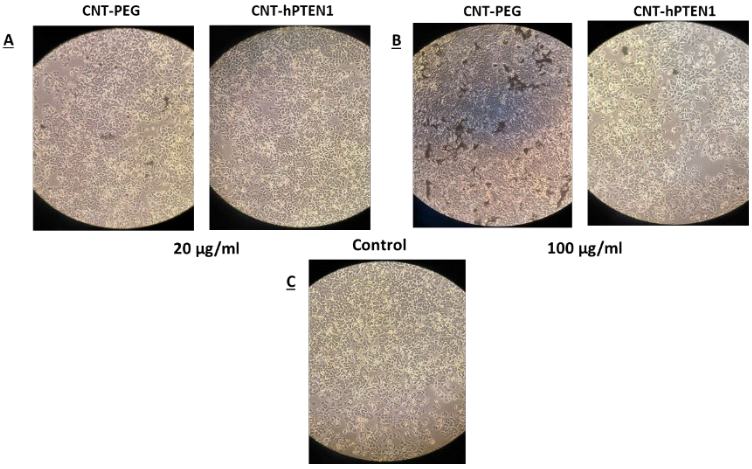

Figure S9. MCF-7 cells morphology after incubation for 72 hours with CNTs-PEG or CNTs-hPTEN1 at a final concentration of 20 µg/ml (**A**), and 100 µg/ml (**B**). Control MCF-7 cells (**C**) also harvested 72 hours after their seeding. Cell photos were taken with a Nikon optical microscope at 100X magnification.

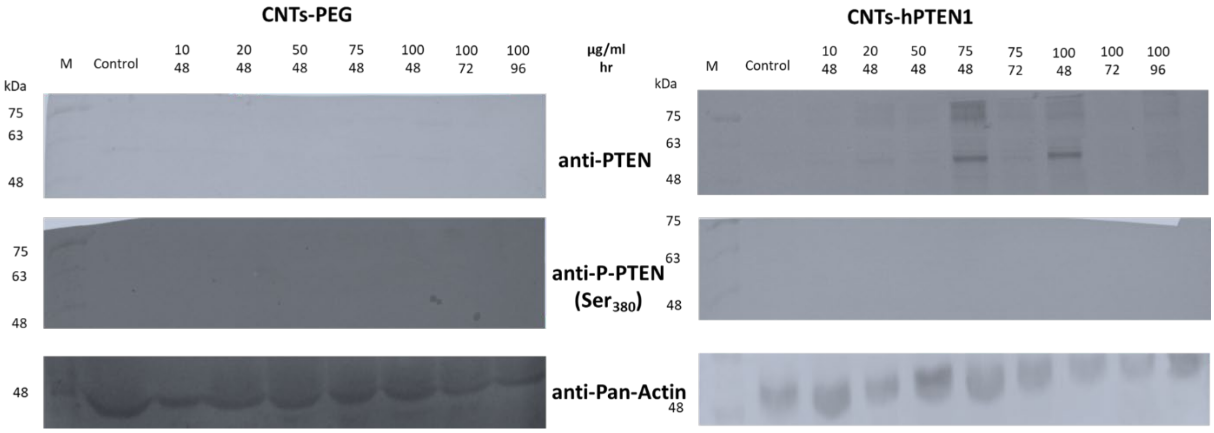

Figure S10. Western blot analysis for 50 µg Control, CNTs-PEG and CNTs-hPTEN1 treated ZR-75-1 cell extracts for anti-PTEN, anti-P-PTEN and anti-Pan-Actin.

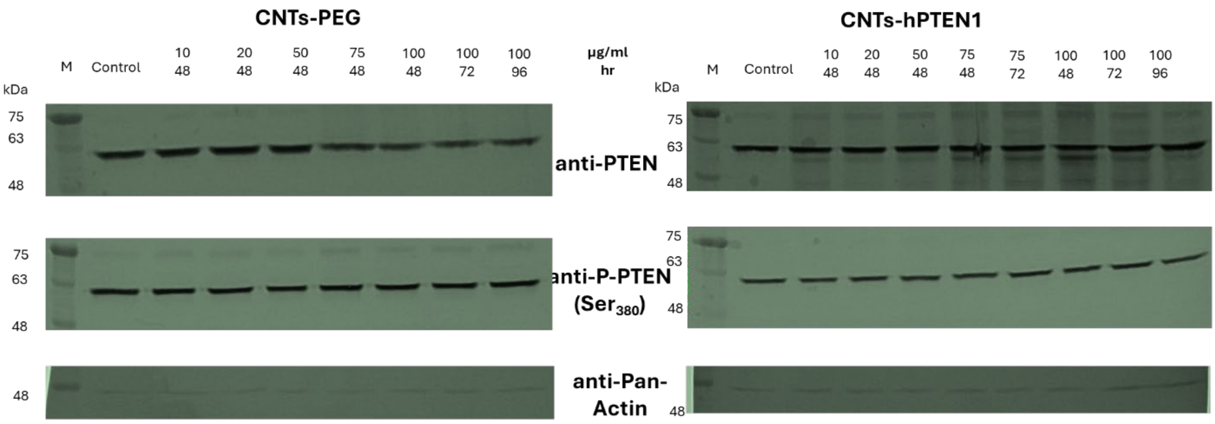

Figure S11. Western blot analysis for 50 μg Control, CNTs-PEG and CNTs-hPTEN1 treated MCF-7 cell extracts for anti-PTEN, anti-P-PTEN and anti-Pan-Actin.

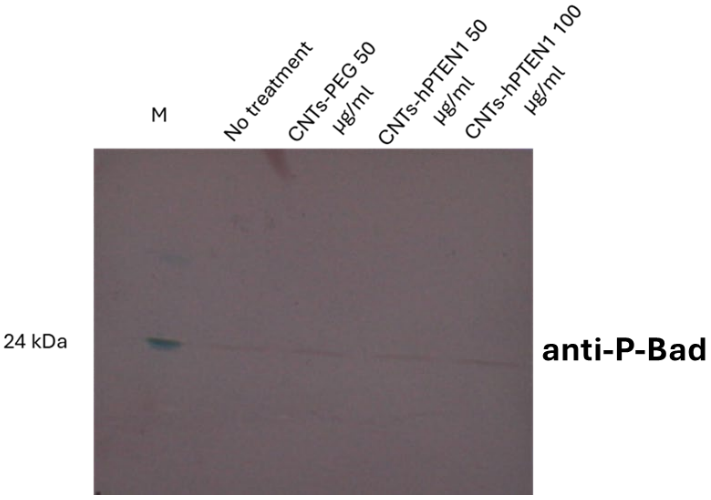

Figure S12. Western blot analysis for anti-P-Bad of 50 μg MCF-7 cell extracts treated for 96 hours with 100 μg/mL of CNTs-PEG, 50 and 100 μg/mL of biofunctionalized CNTs-hPTEN1 and after no treatment (control).

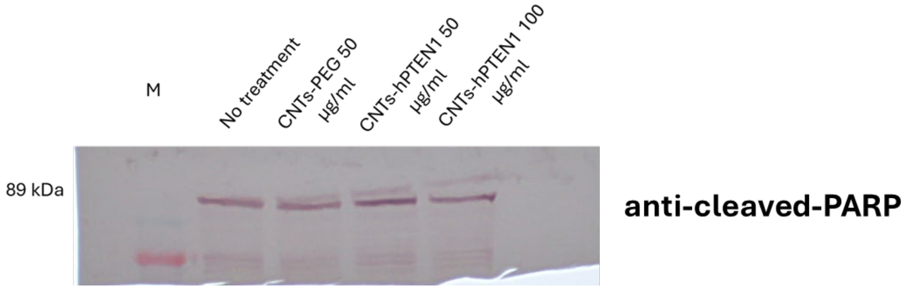

Figure S13. Western blot analysis for anti-cleaved-PARP of 50 μg MCF-7 cell extracts treated for 96 hours with 100 μg/mL of CNTs-PEG, 50 and 100 μg/mL of biofunctionalized CNTs-hPTEN1 and after no treatment (control).
